# Age-Dependent Changes of Monocarboxylate Transporter 8 Availability in the Postnatal Murine Retina

**DOI:** 10.3389/fncel.2016.00205

**Published:** 2016-08-26

**Authors:** Yoshiyuki Henning, Karol Szafranski

**Affiliations:** ^1^Department of General Zoology, Faculty of Biology, University of Duisburg-EssenEssen, Germany; ^2^Genome Analysis, Leibniz Institute on Aging – Fritz Lipmann InstituteJena, Germany

**Keywords:** thyroid hormone, T4, T3, thyroid hormone transporter, MCT8, retina, photoreceptors, mouse

## Abstract

The thyroid hormones (TH) triiodothyronine (T3) and its prohormone thyroxine (T4) are crucial for retinal development and function, and increasing evidence points at TH dysregulation as a cause for retinal degenerative diseases. Thus, precise regulation of retinal TH supply is required for proper retinal function, but knowledge on these mechanisms is still fragmentary. Several transmembrane transporters have been described as key regulators of TH availability in target tissues of which the monocarboxylate transporter 8 (MCT8), a high affinity transporter for T4 and T3, plays an essential role in the central nervous system. Moreover, in the embryonic chicken retina, *MCT8* is highly expressed, but the postnatal availability of MCT8 in the mammalian retina was not reported to date. In the present study, spatiotemporal retinal MCT8 availability was examined in mice of different age. For this purpose, we quantified expression levels of *Mct8* via Real-Time Reverse-Transcriptase PCR in mouse eyecups (C57BL/6) of juvenile and adult age groups. Additionally, age-dependent MCT8 protein levels were quantified via Western blotting and localized via immunofluorescence confocal microscopy. While no difference in *Mct8* expression levels could be detected between age groups, MCT8 protein levels in juvenile animals were about two times higher than in adult animals based on Western blot analyses. Immunohistochemical analyses showed that MCT8 immunoreactivity in the eyecup was restricted to the retina and the retinal pigment epithelium. In juvenile mice, MCT8 was broadly observed along the apical membrane of the retinal pigment epithelium, tightly surrounding photoreceptor outer segments. Distinct immunopositive staining was also detected in the inner nuclear layer and the ganglion cell layer. However, in adult specimens, immunoreactivity visibly declined in all layers, which was in line with Western blot analyses. Since MCT8 was abundantly present in juvenile and about twofold lower in adult retinae, our findings suggest a pivotal role of MCT8 especially during postnatal maturation. The present study provides novel insights into age-dependent retinal TH supply, which might help to understand different aspects regarding retinal development, function, and disorders.

## Introduction

Thyroid hormones, in particular 3,5,3′-triiodothyronine (T3) and its precursor thyroxine (T4), are widely associated with somatic and neuronal development, and metabolism. T3 is a ligand for different THRs, nuclear receptors regulating the expression of a wide range of target genes (Morreale De Escobar et al., 1985; Lavado-Autric et al., 2003; Mullur et al., 2014). The availability of T3 is mostly regulated by intracellular conversion of T4 by specialized enzymes, deiodinases type 1 and 2 (D1, D2). Inactivation of TH is catalyzed by deiodinases type 1 and 3 (D1, D3; Bianco and Kim, 2006). In the mammalian retina, a strict TH regulation was found crucial for photoreceptor development and function (Roberts et al., 2006; Ng et al., 2010; Glaschke et al., 2011; Sawant et al., 2015).

Most mammals possess two types of photoreceptors, rods for dim light vision, and cones for daylight, i.e., color vision. While rods express only one type of opsin (rhodopsin), a light-sensitive molecule enabling phototransduction, mammalian cones usually express short (S) and medium (M) wavelength sensitive opsins, and some primates including humans express an additional long (L) wavelength sensitive opsin (Hunt and Peichl, 2014). In cones, a particular THR isoform, THRβ2, is expressed and was identified as a crucial upstream factor for M/L-opsin expression (Ng et al., 2001; Applebury et al., 2007). While cones first express S-opsins in early postnatal stage (Hunt and Peichl, 2014), binding of T3 to THRβ2 is required for the onset of M/L-opsin expression, and simultaneous suppression of S-opsin (Ng et al., 2001; Roberts et al., 2006; Cheng et al., 2009; Forrest and Swaroop, 2012). Consistent with this mechanism, hypothyroidism leads to reduced M/L-opsin expression even in adult age, which can be restored by TH treatment in rodent models as well as human (Glaschke et al., 2011; Cakir et al., 2015). On the other hand, inactivation of TH by D3 was shown crucial for cone development. In *D3* knockout mice, about 80% of cones are lost by neonatal cell death (Ng et al., 2010). Additional deletion of *Thrβ2* leads to cone protection, suggesting that excessive TH signaling via THRβ2 has a deleterious effect on cones. Similar findings were reported in mouse models of retinal dystrophy where TH suppression (Ma et al., 2014) and D2 inhibition (Yang et al., 2016) have a protective function on cones, while hyperthyroidism leads to cone degeneration (Ma et al., 2014). Moreover, in a prospective study, high serum levels of the prohormone T4 positively correlated with a higher prevalence for age-related macular degeneration, while elevated T3 had no impact (Chaker et al., 2015). The underlying mechanism for TH induced cone degeneration is not known, but TH dysregulation has already been linked to reduced renewal of photoreceptor OS and retinal arteriolar narrowing (Takeda et al., 1996; Ittermann et al., 2014), both processes associated with the pathogenesis of different degenerative diseases. Even in rods, involvement of TH signaling has been associated with development and function (Ng et al., 2010; Sawant et al., 2015).

While TH were shown to be essential for photoreceptor development and function, the mechanisms regulating retinal TH supply are still poorly understood. Nourishment of photoreceptors is regulated through the RPE, a monolayer of pigmented cells with its apical membrane lying adjacent to photoreceptor OS. The photoreceptor OS are tightly surrounded by microvilli emerging from the RPE, maintaining a complex of close interaction (Strauss, 2005). The RPE builds a part of the outer BRB, and TH, as many other organic compounds, are transported via transmembrane transporters from the choriocapillaris into photoreceptors and vice versa. Unlike photoreceptors, interneurons (bipolar cells, horizontal cells, and amacrine cells), and ganglion cells located in the inner retinal layers are not connected to the RPE. Nourishment of these layers is thus facilitated through retinal capillaries found in the layers between the OPL and the GCL. Endothelial cells, pericytes, and Müller cells (the most prominent retinal glial cells) build up the inner BRB which regulates nourishment of inner retinal cells (Hosoya and Tachikawa, 2012).

Several transporters have been described which facilitate the influx and/or eﬄux of TH across plasma membranes as primary or secondary substrates in diverse tissues (reviewed in Kinne et al., 2011; Bernal et al., 2015). The MCT8 (encoded by *Slc16a2*, hereafter: *Mct8*) is a high affinity TH transporter expressed in several tissues (Pizzagalli et al., 2002; Friesema et al., 2003; Sugiyama et al., 2003; Bernal et al., 2015). MCT8 transports T4 as well as T3, with a higher affinity for T3 (Friesema et al., 2003), and is highly expressed in diverse tissues (Kinne et al., 2011). In the murine brain, *Mct8* is highly expressed during the first postnatal weeks (Müller and Heuer, 2014), where it possesses a critical role in TH uptake into the brain (Ceballos et al., 2009; Visser et al., 2011), especially into neurons (Friesema et al., 2005). TH signaling is required for proper brain development and hypothyroidism in the critical phase of postnatal brain maturation in rodents (e.g., neuronal differentiation, dendritic branching, axon growth and synaptogenesis) leads to drastically diminished neuronal connectivity in rodents (Horn and Heuer, 2010). In the embryonic chicken retina, *MCT8* mRNA is widely expressed (Geysens et al., 2012; Bourgeois et al., 2016). To our knowledge no data is available regarding MCT8 availability in the postnatal vertebrate eye, although MCT8 is one promising candidate for regulation of retinal TH supply to maintain proper retinal maturation and function like in the brain. In order to contribute to a deeper understanding of retinal TH supply, we quantified retinal *Mct8* mRNA expression by means of quantitative reverse transcriptase-mediated polymerase chain reaction (qRT-PCR). Protein levels and localization of MCT8 were further investigated by Western blotting and immunohistochemistry, respectively. We focused on juvenile and adult life stages where TH dysregulation has major impact on cone opsin expression and photoreceptor viability.

## Materials and Methods

### Animals

Male mice (C57BL/6) aged 14 days (P14; juvenile), 21 days (P21; juvenile), 28 days (P28; juvenile), 24 weeks (6M; adult), and 24 months (24M; old) were obtained from the Department of Developmental Biology (University of Duisburg-Essen, Essen, Germany) and the Central Animal Laboratory, University Hospital Essen. The three juvenile stages are representative for the onset of vision (P14; Sharma et al., 2003; Dorrell et al., 2004), terminal differentiation of retinal structures (P21, e.g., synaptogenesis, pruning, and maturation of vascular pattern; Dorrell et al., 2004; Banday et al., 2014), and a transitional stage between juvenile and adult mice (P28; i.e., onset of puberty; Berry and Bronson, 1992). 6 and 24M mice represent adult stages, from which 6M retinae can be considered fully mature, and 24M specimen represent aging retinae (Berry and Bronson, 1992).

Mice were housed in standard macrolone cages and were fed *ad libitum* with commercial food pellets. Especially in the 24M group, the animals were checked for obvious degenerative changes of the eye, such as cataracts. For all experimental procedures mice were deeply anesthetized with isoflurane (AbbVie, Wiesbaden, Germany), and subsequently sacrificed by cervical dislocation. Maintenance and all treatments of the animals reported here are in agreement with the North Rhine-Westphalia State Environment Agency.

### RNA Preparation and qRT-PCR

Freshly isolated eyes (*n* = 6 per age group) were incised at the corneal rim to remove the cornea, lens, and vitreous. The eyecup was transferred to stabilization reagent RNA*later* (Qiagen, Hilden, Germany), incubated 24 h at room temperature, as recommended, and stored at -20°C for later expression analyses. Additionally, temporal neocortex including the cerebral membrane was isolated and conserved in RNA*later*.

For RNA extraction, the eyecups were immersed into lysis buffer (Buffer RLT, Qiagen, Hilden, Germany) and homogenized with a TissueLyser II (Qiagen). Total RNA was extracted by RNeasy Mini Kit (Qiagen) according to manufacturer’s instructions. Complementary DNA (cDNA) was synthesized using AMV Reverse Transcriptase (Promega; catalog no. A3500) primed by random oligomers. We reverse-transcribed 0.25 μg RNA in 20-μl reactions, as recommended by the manufacturer’s instructions, albeit with the addition of 10 μg bovine serum albumin per reaction. We selected three reference genes, *Hprt, Atp2b4*, and *Mapk1*, in order to obtain normalized mRNA expression data. All these genes showed stable expression levels in previous studies of mammalian retina across different postnatal life stages (Loeﬄer et al., 2002; Rocha-Martins et al., 2012; Adachi et al., 2015). Furthermore, these genes encode proteins with different functional roles and subcellular locations. The validity of this concept was also supported by cross-correlation of the reference genes’ RT-PCR Ct values in this study, which did not reveal any age-related dependencies (results not shown). DNA primer pairs (metabion international, Planegg, Germany) used for qRT-PCR were: 5′-TCC CAT TGC ATT TGA GCT G-3′ and 5′-GGG ACA CCC GCA AAG TAG A-3′ (*Mct8*), 5′-GCT GGT GAA AAG GAC CTC TC-3′ and 5′-CAA GGG CAT ATC CAA CAA CA-3′ (*Hprt*), 5′-AAC TCA GTG CGC AAG TCC AT-3′ and 5′-TCC TTC CTT GTT CAG GAT TCG-3′ (*Atp2b4*), 5′-CTC TGG CCC ACC CAT ACC T-3′ and 5′-AAG TCG TCC AAC TCC ATG TCA-3 (*Mapk1*). Real-time qRT-PCR was performed using SybrGreen (Invitrogen, Carlsbad, CA, USA) according to the manufacturer’s instructions. The thermal cycler (iQ Cycler, Bio-Rad, Hercules, CA, USA) was programmed to initial 95°C for 90 s and 40 cycles comprising 95°C for 45 s, 55°C for 25 s, and 72°C for 30 s. Each assay, defined by sample/primer combination, was performed in triplicate of independent qPCR reactions, and the Ct values were averaged before normalization. Normalized expression levels were obtained according to the ΔΔ*C*t method, accounting for primer-specific amplification efficiencies (Liu and Saint, 2002), which were determined from a four-step dilution series of an arbitrary cDNA sample.

The data was kept in *C*t scale (log_2_) for a statistical analysis using one-way ANOVA followed by Tukey’s multiple comparison test using GraphPad Prism (GraphPad Software Inc., San Diego, CA, USA), differences being considered significant when *p* < 0.05.

### Antibodies

For the detection of MCT8 protein we used a commercial rabbit polyclonal IgG (HPA003353, Sigma-Aldrich, Taufkirchen, Germany; dilution Western Blot 1:1000, immunohistochemistry 1:500). For Western blotting, we used a rabbit polyclonal H3 IgG (ab1791, Abcam, Cambridge, UK; dilution 1:5000) as internal loading control. A horseradish peroxidase-conjugated swine anti-rabbit IgG (P0217, Dako, Hamburg, Germany; dilution 1:10000) was used as a secondary antibody. For immunohistological analyses, we labeled S-opsin with a goat polyclonal IgG (sc-14363, Santa Cruz Biotechnology, Dallas, TX, USA; dilution: 1:500), and Glucose transporter 1 (GLUT1) with a mouse monoclonal antibody (ab40084, Abcam; dilution 1:100), to enable a better localization of MCT8 immunostaining in retinal cryosections. To visualize MCT8, GLUT1, and S-opsin in each section, sections were incubated with a mixture of the three primary antibodies, and three secondary antibodies with well separated excitation and emission spectra were chosen. Binding sites of the anti-MCT8 antibody were revealed by Alexa Fluor 647 donkey anti-rabbit (false colored in red), anti-S-opsin by Alexa Fluor 568 donkey anti-goat (false colored in cyan), and anti-GLUT1 antibodies by Alexa Fluor 488 donkey anti-mouse (false colored in green), at a dilution of 1:500 (Abcam).

### Western Blotting

After enucleation, eyecups were transferred to ice cold lysis buffer (20 mM Tris pH 8.0, 1 mM EDTA, 5 mM MgCl_2_, 1 mM DTT, 1% Triton X-100, and 1% protease inhibitor) and homogenized using a Potter-Elvehjem homogenizer. Samples (*n* = 4 per age group) were centrifuged (11 min, 13,000 rpm, 4°C) and the protein concentration in the supernatants was determined by a biuret protein assay (Roti-Quant Universal, Carl Roth, Karlsruhe, Germany). For protein separation, 1x Laemmli buffer was added to the samples, vortexed for 5 s and put back on ice. Proteins were separated on an 8% SDS-polyacrylamide gel by applying 15 μg total protein of each sample, and transferred to a polyvinylidene difluoride (PVDF) membrane.

Membranes were blocked with blocking buffer (5% skimmed milk in 0.1% TBS-T) for 1 h at room temperature and incubated overnight at 4°C with rabbit anti-MCT8 antibody and rabbit anti-H3 antibody (internal loading control) diluted in the same blocking buffer (see antibody section for details). We selected H3 for normalization, since other familiar loading controls such as tubulin, actin, or GAPDH are not expressed uniformly across age groups (Rocha-Martins et al., 2012; Taylor and Posch, 2014). H3 is expressed at stable levels in the postnatal mouse retina (Banday et al., 2014), and as a nuclear protein, it additionally allowed validation of efficient dissolution of plasma membranes. Membranes were washed three times in 0.1% TBS-T and incubated with horseradish peroxidase-conjugated swine anti-rabbit secondary antibody for 1 h at room temperature. Binding sites were visualized by means of a chemiluminescence detection kit (AceGlow, VWR International, Langenfeld, Germany). The signal densities obtained for each band were quantified with Bio-1D advanced (Vers. 12.11, Vilber Lourmat, Eberhardzell, Germany), and relative density values were calculated with the first sample set as reference. Relative MCT8 expression was then normalized with relative H3 of respective samples, and statistically analyzed with one-way ANOVA followed by Tukey’s multiple comparison test using GraphPad Prism (GraphPad Software Inc., San Diego, CA, USA).

### Immunohistochemical Analysis

Whole eyes were fixed in 4% paraformaldehyde in 0.1 M phosphate buffer (PB; pH 7.4) at 4°C for 3 h. For further processing, cornea, lens, and vitreous were removed carefully. Then, eyecups were immersed in a successive series of 10, 20, and 30% sucrose in PB for cryoprotection, mounted in Tissue-Tek O.C.T. (Sakura Finetek Germany, Staufen, Germany), and snap-frozen in 2-methylbutane cooled in liquid nitrogen. Frozen sections were cut (sagittal, 16 μm) using a cryostat (CM3000, Leica Biosystems), mounted on silanized glass slides (SuperFrost Ultra Plus, Thermo Fisher Scientific, Carlsbad, CA, USA) and air-dried for 24 h. The sections were washed in PB, and blocked with 10% donkey serum (Sigma-Aldrich, Taufkirchen, Germany) in PB with 1% BSA and 0.5% Triton X-100 (Carl Roth) for 1 h at room temperature. Incubation with primary antibodies (mixture of anti-MCT8, anti-S-opsin, and anti-GLUT1; see antibody section for details) was performed overnight at 4°C in 3% donkey serum with 1% BSA and 0.5% Triton X-100 in PB. After washing in PB, Alexa Fluor conjugated secondary antibodies, appropriately diluted in the same buffer as used for primary antibody, were incubated for 1 h at room temperature. Sections were washed in PB and coverslipped with Roti-Mount FluorCare DAPI (Carl Roth) for supplemental nuclei staining.

Structures labeled by immunofluorescence were visualized using a confocal laser scanning microscope (Zeiss ELYRA PS.1 super resolution microscope combined with a LSM710) equipped with a 405 nm diode laser, an Argon Multiline 458/488/568 and a 633 nm Helium-Neon Laser. Micrographs were captured by means of confocal software (ZEN system 2012 Black Edition, Zeiss), and adjusted for brightness and contrast only.

## Results

### Mct8 Expression

**FIGURE 1:**
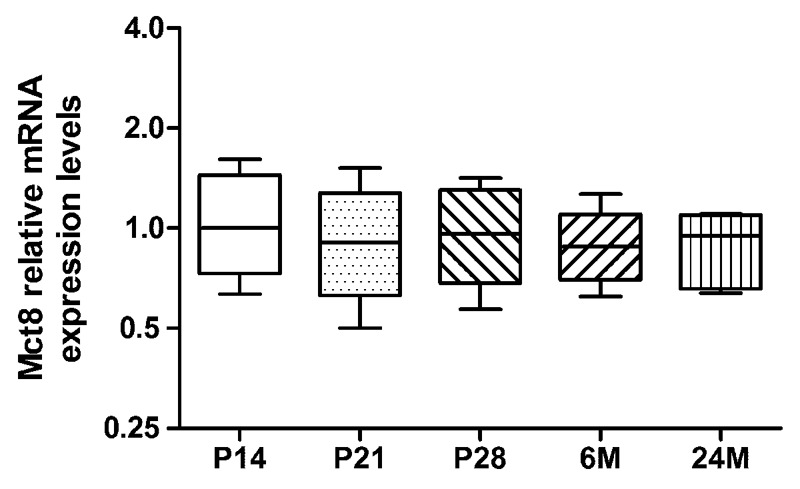
**Expression levels of *Mct8* mRNA: quantitative reverse transcriptase-mediated polymerase chain reaction (qRT-PCR) was performed with eyecup homogenates at varying age (P14, P21, P28, 6M, and 24M).** *Mct8* mRNA levels are given as fold-changes relative to P14 expression levels, arbitrarily set to 1. Note that normalization and statistical analysis were performed in the *C*t scale (log_2_), as described in “Materials and Methods.” Data are presented as fold changes, median ±25%/75% quartile and maximum/minimum value (*n* = 6 per age group).

We applied qRT-PCR in order to obtain an overview of *Mct8* mRNA expression in the mouse eyecup at different ages. Statistical analysis of *Mct8* levels revealed no significant differences between the tested age groups (one-way ANOVA, *F* = 0.19, *p* = 0.94, **Figure 1**), while the age-matched, inter-individual variation was about 1.4-fold. This result was independent of the normalization procedure since normalization to total RNA input produced the same qualitative test results as did normalization to reference genes. Thus, qRT-PCR results suggest that *Mct8* is expressed at almost equal levels in murine eyecups of different age.

### Western Blot Analysis

**FIGURE 2:**
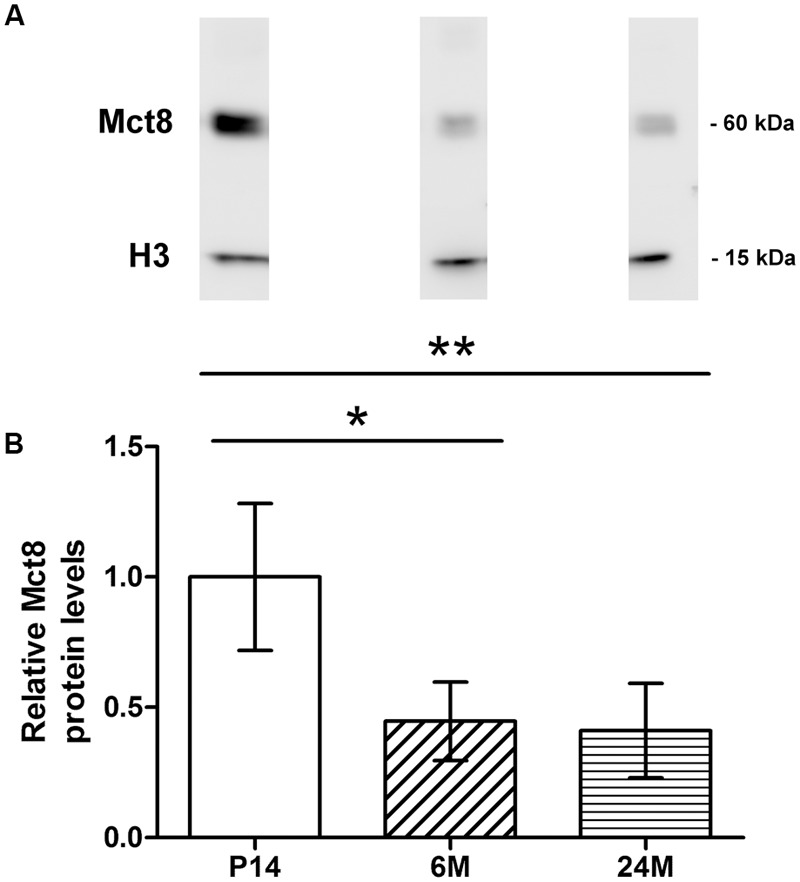
**Western blot analysis of MCT8: Western blotting was performed with eyecup homogenates of P14, 6M, and 24M mice with an antibody against MCT8 (predicted: 60 kDa) and an antibody against H3 (predicted: 15 kDa) as internal loading control.** Each blot **(A)** corresponds to the age groups shown in the barplot below. **(B)**. Each band is representative for one age group and was taken from the same blot, respectively. Barplots are depicted relative to P14 MCT8 protein levels, arbitrarily set to 1. Specific bands for MCT8 and H3 were detected in the Western blots, with significant differences between P14 and 6M/24M. Data are presented as mean ± SD (*n* = 4 per age group). ^∗∗^*p* < 0.005; ^∗^*p* < 0.05.

To assess possible differences in MCT8 protein levels, we quantified protein levels in three experimental groups representing three life stages (P14: juvenile; 6M: adult; 24M: old), by means of Western blotting with eyecup homogenates (15 μg protein). The anti-MCT8 antibody recognized a single band in all samples with an apparent size of 60 kDa, which is consistent with the predicted protein size and previous reports (Friesema et al., 2003; Roberts et al., 2008) (**Figure 2A**; Supplementary Figure S1 for complete gel). Anti-H3 antibody, used for normalization, recognized a single band in each sample with an apparent size of 15 kDa (predicted: 15 kDa) (**Figure 2A**; Supplementary Figure S1).

Mean normalized MCT8 protein levels were more than twofold higher in P14 compared to 6 and 24M, and revealed significant differences between the juvenile and both adult age groups in the statistical analysis (one-way ANOVA: *F* = 9.71, *p* = 0.0057; Tukey’s multiple comparison test: P14 vs. 6 M: *p* < 0.05; P14 vs. 24M: *p* < 0.005; **Figure 2B**). In contrast, no significant difference was found between 6 and 24M protein levels.

Liver homogenates were used as positive control for the MCT8-antibody, which revealed a broad band with an apparent size of 60 kDa (Supplementary Figure S2).

### Immunohistochemical Localization of MCT8 in the Retina

We performed immunohistological stainings on eyecup cryosections of five age groups (P14, P21, P28, 6M, and 24M) to determine MCT8 localization in the mouse retina throughout different postnatal life stages. GLUT1 and DAPI were co-stained to control for normal retinal morphology. Moreover, GLUT1 staining was raised to identify the basolateral and apical RPE membrane, since GLUT1 is localized at both membranes and RPE cells can be very thin especially in juvenile animals.

**FIGURE 3:**
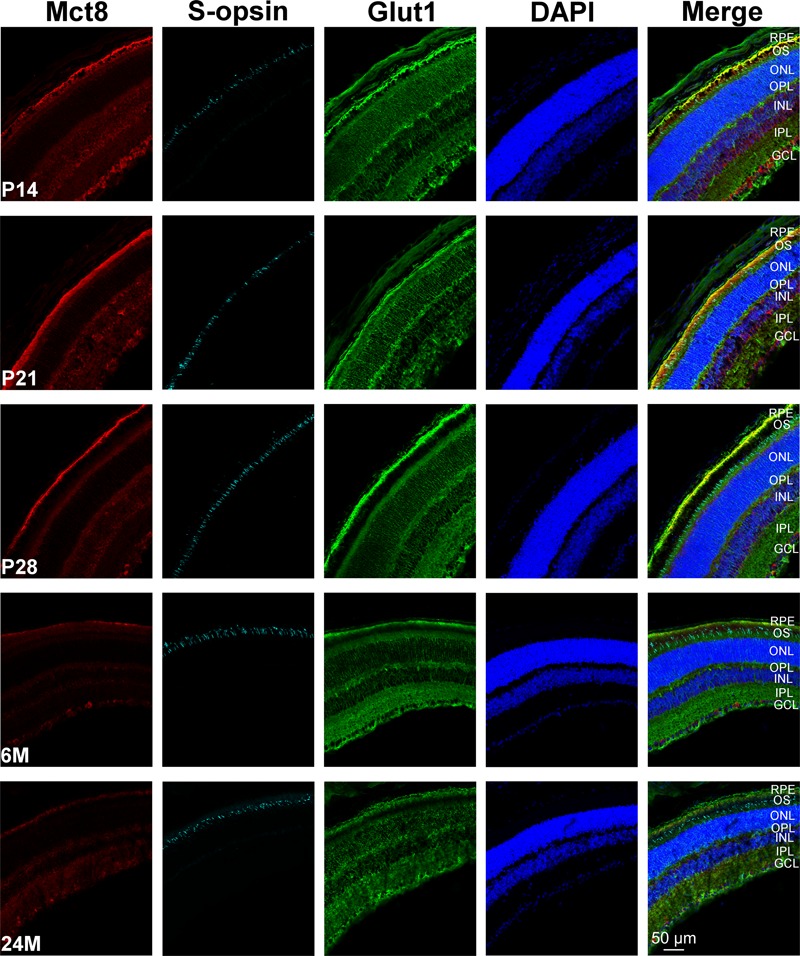
**Overview of MCT8 immunoreactivity in the mouse retina: Eyecup cryosections of five age groups (P14, P21, P28, 6M, 24M) colabeled with antibodies against MCT8 (red), GLUT1 (green), S-opsin (cyan) are shown.** Nuclei were counterstained with DAPI (blue). Antibody binding sites were revealed using Alexa Fluor conjugated secondary antibodies and visualized with a Zeiss Elyra PS.1 combined with a LSM710 confocal microscope.

**FIGURE 4:**
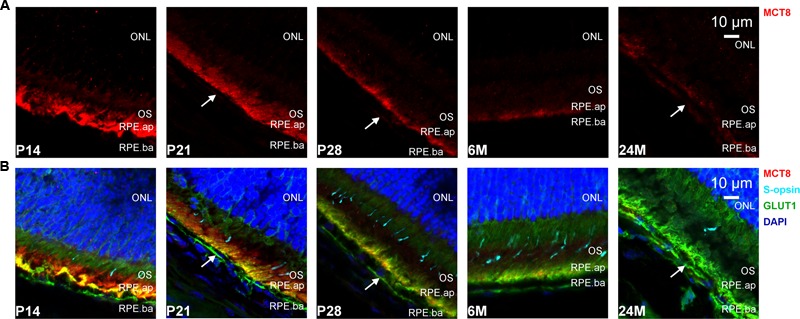
**MCT8 immunoreactivity in the mouse RPE: Eyecup cryosections colabeled with antibodies against MCT8 (red), GLUT1 (green), S-opsin (cyan) are shown.** Nuclei were counterstained with DAPI (blue). Antibody binding sites were revealed using Alexa Fluor conjugated secondary antibodies and visualized with a Zeiss Elyra PS.1 combined with a LSM710 confocal microscope. **(A)** MCT8 immunoreactivity in the RPE apical membrane (RPE.ap) and basolateral membrane (RPE.ba). **(B)** Merged images are shown for better localization of MCT8 in the respective retinal layers. Predominant staining was observed in RPE.ap, whereas RPE.ba showed only faint and irregular immunoreactivity throughout all age groups (white arrows). The signal declined gradually in the apical membrane, with the strongest signal observed in P14 and P21, and only scarcely distributed in 24M. In OS and ONL no specific staining was observed in all age groups.

**FIGURE 5:**
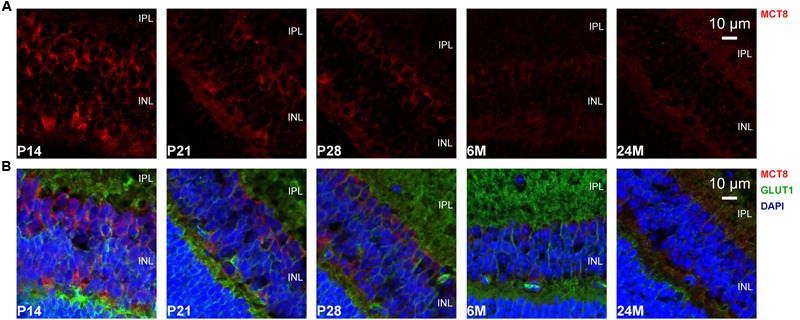
**MCT8 immunoreactivity in the mouse INL: Eyecup cryosections colabeled with antibodies against MCT8 (red) and GLUT1 (green) are shown.** Nuclei were counterstained with DAPI (blue). Antibody binding sites were revealed using Alexa Fluor conjugated secondary antibodies and visualized with a Zeiss Elyra PS.1 combined with a LSM710 confocal microscope. **(A)** MCT8 immunoreactivity in the INL. **(B)** Merged images are shown for better localization of MCT8 in the respective retinal layers. MCT8 was localized in plasma membranes of cell bodies located in the INL. The signal intensities gradually declined, with the strongest signal observed in P14, and only faintly detectable in 24M. In the IPL no specific staining was observed in all age groups.

**FIGURE 6:**
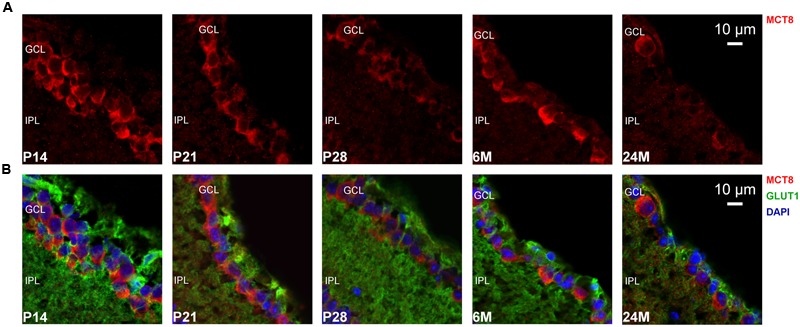
**MCT8 immunoreactivity in the mouse GCL: Eyecup cryosections colabeled with antibodies against MCT8 (red) and GLUT1 (green) are shown.** Nuclei were counterstained with DAPI (blue). Antibody binding sites were revealed using Alexa Fluor conjugated secondary antibodies and visualized with a Zeiss Elyra PS.1 combined with a LSM710 confocal microscope. **(A)** MCT8 immunoreactivity in the GCL. **(B)** Merged images are shown for better localization of MCT8 in the respective retinal layers. MCT8 was localized in plasma membranes of cell bodies located in the GCL. The signal intensities gradually declined with the strongest signal observed in P14, and only detectable around single nuclei in 24M. In the IPL no specific staining was observed in all age groups.

The overall retinal morphology was intact in all age groups, showing the typical layering known from mammalian retinae (RPE; OS; ONL; OPL; INL; IPL; GCL). However, MCT8 immunoreactivity showed apparent age-dependent changes: In all age groups, MCT8 immunoreactivity was observed in the RPE, and around nuclei of the INL and the GCL in the whole retina, but the intensity visibly declined from juvenile to adult stages (**Figures 3–6**). Detailed inspection of MCT8 immunoreactivity in the RPE revealed that MCT8 is predominantly located at the apical membrane (RPE.ap) with its microvilli, while the basolateral membrane (RPE.ba) showed only faint and irregularly distributed immunopositive signal in all age groups (**Figure 4**; white arrows). At the RPE.ap of P14 and P21 specimens, MCT8 immunoreactivity reached deep into photoreceptor OS indicated by S-opsin staining, while a slight decline in immunoreactivity was already visible in the RPE.ap of P28 specimens (**Figure 4**). The decline of MCT8 was even more pronounced in the 6 and 24M retinae, where immunoreactivity in the RPE.ap was restricted to the surface except of short and irregular branches (**Figure 4**). In the INL and GCL, MCT8 was found at plasma membranes of cell bodies, indicated by nuclei staining (**Figures 5** and **6**). While in P14, a vast majority of cell bodies showed positive MCT8 staining, the immunoreactivity gradually declined, and was only scattered around single nuclei in the INL and GCL of adult animals.

Overall, MCT8 immunoreactivity in the mouse retina was strongest during the phase of retinal maturation and showed a gradual decline in adult stages. A difference between the ventral and dorsal retina was not observed in the micrographs, despite of a dorsoventral S-opsin gradient typical for this mouse species (not shown).

A negative control in which we omitted the primary antibodies, showed no staining despite of some faint autofluorescence in the sclera and RPE.ba in the GLUT1 channel (green; Supplementary Figure S3). Furthermore, a positive control in which we stained brain cryosections with the same anti-MCT8 antibody was performed. Immunoreactivity was found around neuronal cell bodies similar to the staining observed in the retinal ganglion cells (Supplementary Figure S4).

## Discussion

Previous studies showed that TH are crucial for photoreceptor development and function in vertebrates, including human (Ng et al., 2001; Roberts et al., 2006; Cheng et al., 2009; Glaschke et al., 2011; Cakir et al., 2015; Sawant et al., 2015), and recent studies even showed that TH dysregulation could be involved in photoreceptor degeneration such as age-related macular-degeneration (Ma et al., 2014; Chaker et al., 2015). In the present study, we investigated the availability of MCT8, known as an essential TH transporter of the nervous system, in the postnatal murine retina with focus on age-dependent changes.

We could show that MCT8 protein is localized in the RPE, INL, and GCL of all age groups, but we found an age-dependent decrease of MCT8 levels between juvenile and adult age groups. However, on mRNA level no significant differences between age groups were found for *Mct8*, suggesting that *Mct8* is expressed at almost equal levels throughout life. This is different to findings in the murine brain where *Mct8* mRNA was reported to decline with age (Müller and Heuer, 2014). Although we homogenized whole eyecups containing RPE and retina for expression analyses and Western blotting, we assume that our data represent MCT8 mRNA and protein levels of the RPE and retina solely, because MCT8 immunoreactivity was not detected in any other eyecup layer by immunohistochemistry, suggesting an absence in the choroid and the sclera lying posterior to the RPE (**Figures 3** and **4**).

Constant *Mct8* expression in combination with decreasing protein levels might be contradictory, but this kind of asymmetry between MCT8 mRNA and protein levels was previously reported in liver and kidney as well (Silvestri et al., 2008). In the study by Silvestri et al. (2008), *Mct8* mRNA of 24M mice exceeded the levels of younger age groups by about 52%, while MCT8 protein levels decreased. In the kidney no age-dependent changes in MCT8 protein levels were observed, but mRNA levels decreased in old animals. Taken together, *Mct8* mRNA expression and protein turnover seems to follow a non-linear pattern in diverse tissues. In the retina, excessive TH signaling was reported to have deleterious effects on photoreceptor viability (Ng et al., 2010; Ma et al., 2014), hence downregulation of MCT8 protein levels could be interpreted as a post-transcriptional mechanism to avoid oversupply of TH sensitive photoreceptor cells. A similar protective mechanism was reported for the TH inactivating enzyme D3 in the embryonic mouse retina. *D3* deletion leads to neonatal degeneration of immature cones, while additional *Thrβ2* deletion prevents early cone loss, suggesting that D3 has a protective function in the immature retina by inhibiting TH signaling via THRβ2 (Ng et al., 2010). However, *D3* mRNA levels and activity decline rapidly after birth, suggesting that TH signaling contributes to postnatal processes involved in retinal maturation. These processes include differentiation and cell fate determination of retinal precursor cells, axonal growth, M/L-opsin expression, vascularization, and synaptogenesis to form the plexiform layers (Sharma et al., 2003; Dorrell et al., 2004; Banday et al., 2014). In the brain, axonal growth and synaptogenesis are dependent on proper postnatal TH supply, and impaired TH signaling leads to drastically decreased neuronal connectivity (Horn and Heuer, 2010). In the mammalian brain as well as in the retina, many first and second order TH responsive gene products are involved in neuronal maturation, function, and plasticity, such as laminins involved in astrocyte migration and vascularization (Farwell and Dubord-Tomasetti, 1999; Gnanaguru et al., 2013), the neurotrophins BDNF and NT-3 (Giordano et al., 1992; Bennett et al., 1999; Sui et al., 2010), or Reelin involved in patterning of synaptic connectivity (Rice et al., 2001; Sui et al., 2010). Moreover, T3 is required to inhibit S-opsin expression and activate M/L-opsin expression (Roberts et al., 2006; Glaschke et al., 2011) which shows a peak at P21 and is still expressed at high levels in adulthood (Dorrell et al., 2004). TH can even promote angiogenesis by upregulating several pro-angiogenic factors, such as vascular endothelial growth factor (VEGF), basic fibroblast growth factor (bFGF) and angiopoietin 1 (Davis et al., 2009; Savinova et al., 2011). VEGF is produced in RPE cells and has its peak expression in the mouse retina at P21, which overlaps with vascularization processes (Dorrell et al., 2004; Strauss, 2005). Interestingly, T3 signaling leads to an upregulation of angiopoietin 1, while the antagonistic angiopoietin 2 is downregulated (Savinova et al., 2011). T4 was also shown to initiate angiogenesis via a non-genomic signaling pathway (Liu et al., 2014). These pro-angiogenic effects could also play a role in retinal degenerative diseases. For instance, human patients with high T4 levels have a higher risk to develop an age-related macular degeneration via an unknown mechanism, while elevated T3 levels had no effect on its prevalence (Chaker et al., 2015). Interestingly, one form of age-related macular degeneration is characterized by choroidal neovascularization, which could be attributed to T4 induced angiogenesis. Accordingly, T4 induced neovascularization could be addressed to investigate the pathology of age-related macular degeneration in future studies.

MCT8, as a high affinity TH transporter, could thus be a crucial regulator of TH supply in the first postnatal weeks where many TH dependent processes are upregulated. To avoid degenerative effects in the retina associated with TH oversupply (e.g., excessive choroidal neovascularization), the age-dependent decrease of MCT8 protein levels could represent a protective mechanism.

However, a detailed evaluation of MCT8 immunoreactivity in the RPE (**Figure 4**) revealed that MCT8 is mainly found at the apical membrane, particularly in the interface between RPE and photoreceptor OS. Therefore, transport of TH through the basolateral membrane, i.e., from the choriocapillaris into the RPE, is likely to be mediated by other transporters in juvenile as well as adult animals. Similar distribution was also reported for two T4 transporters in the adult rat retina, the OATP1C1 and 1A4 (Gao et al., 2002; Akanuma et al., 2013), but in contrast to MCT8, these two transporters are also evenly distributed along the RPE.ba and endothelial cells of retinal capillaries. Therefore, these two transporters are likely candidates for T4 uptake across the basolateral membrane and endothelial cells of retinal capillaries. However, OATP1C1 and 1A4 were only studied in the retina of adult rats, thus age-dependent analyses are necessary to draw further conclusions. Cell bodies in the INL and GCL also showed strong MCT8 immunoreactivity in the juvenile age groups, which can be explained by the TH dependent postnatal processes involved in retinal maturation stated above (e.g., synaptogenesis and vascularization). Since MCT8 levels decrease after this postnatal phase of maturation, low levels of MCT8 might be sufficient to maintain TH supply in the adult retina. Furthermore, other TH transporters with a lesser affinity might be involved in retinal TH supply, as well.

## Conclusion

The present study along with previous studies suggest that TH transporters in the mouse retina are likely to exhibit a spatiotemporal expression pattern, similar to the mouse brain. The most likely interpretation is a change in retinal TH demand in different life stages. Our findings support a pivotal role of MCT8 in TH supply of photoreceptors, interneurons, and ganglion cells especially during postnatal maturation. However, the primary transport of TH through the outer and inner BRB is likely to be facilitated by transporters other than MCT8. MCT8 was instead localized at transitional zones between the BRB (RPE.ap) and photoreceptor OS, and at the surface of cell bodies (INL, GCL). Thus far, our results along with previous findings suggest a complex network of TH transporters and other TH regulating components in the retina, therefore further investigation is needed to create a scaffold of retinal TH signaling for a deeper understanding of retinal development and function, as well as age-related disorders.

## Author Contributions

Conception of the work: YH. YH and KS contributed to the experimental procedures, data analyses, and drafting of the final manuscript version in equal parts.

## Conflict of Interest Statement

The authors declare that the research was conducted in the absence of any commercial or financial relationships that could be construed as a potential conflict of interest.

## Abbreviations

BRB: blood-retinal barrier
D2: deiodinases type 2
D3: deiodinases type 3
GCL: ganglion cell layer
INL: inner nuclear layer
IPL: inner plexiform layer
MCT8: monocarboxylate transporter 8
OATP: organic anion transporting polypeptide
ONL: outer nuclear layer
OPL: outer plexiform layer
OS: outer segments
RPE: retinal pigment epithelium
RPE.ap: RPE apical membrane
RPE.ba: RPE basolateral membrane
TH: thyroid hormone
THR: thyroid hormone receptor
T3: 3,5,3′-triiodothyronine
T4: thyroxine
