# Supplementary figures and images for: Age-Dependent Changes of Monocarboxylate Transporter 8 Availability in the Postnatal Murine Retina
Source: Front Cell Neurosci. 2016 Aug 26;10:205. doi: 10.3389/fncel.2016.00205 (PMC4999454; doi:10.3389/fncel.2016.00205)

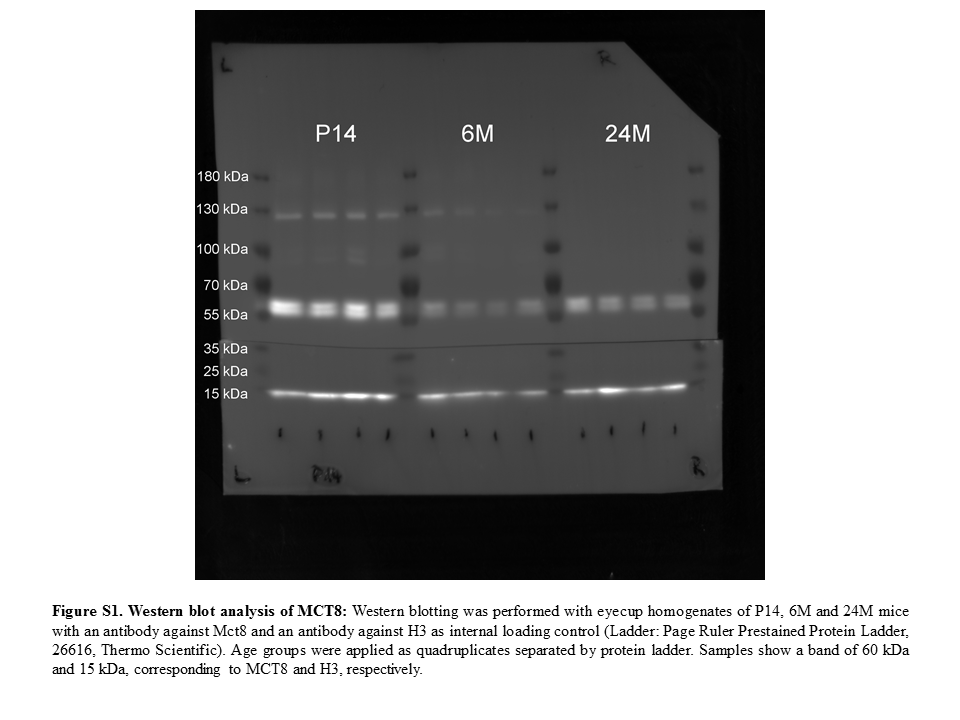

Supplement: Supplementary file 1 [file Image_1.TIF]

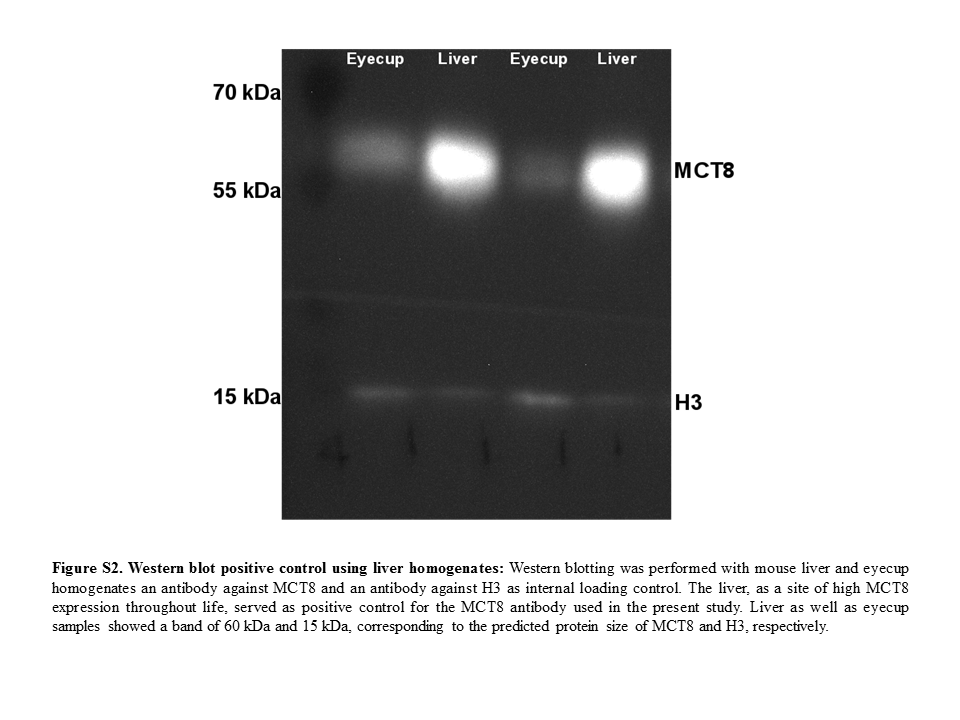

Supplement: Supplementary file 2 [file Image_2.TIF]

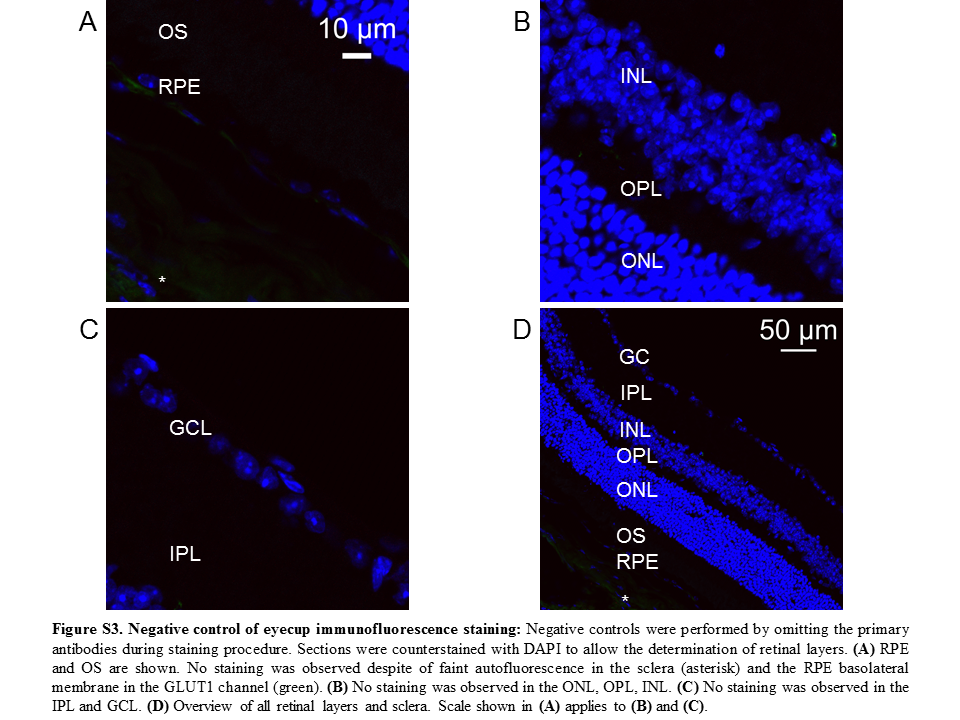

Supplement: Supplementary file 3 [file Image_3.TIF]

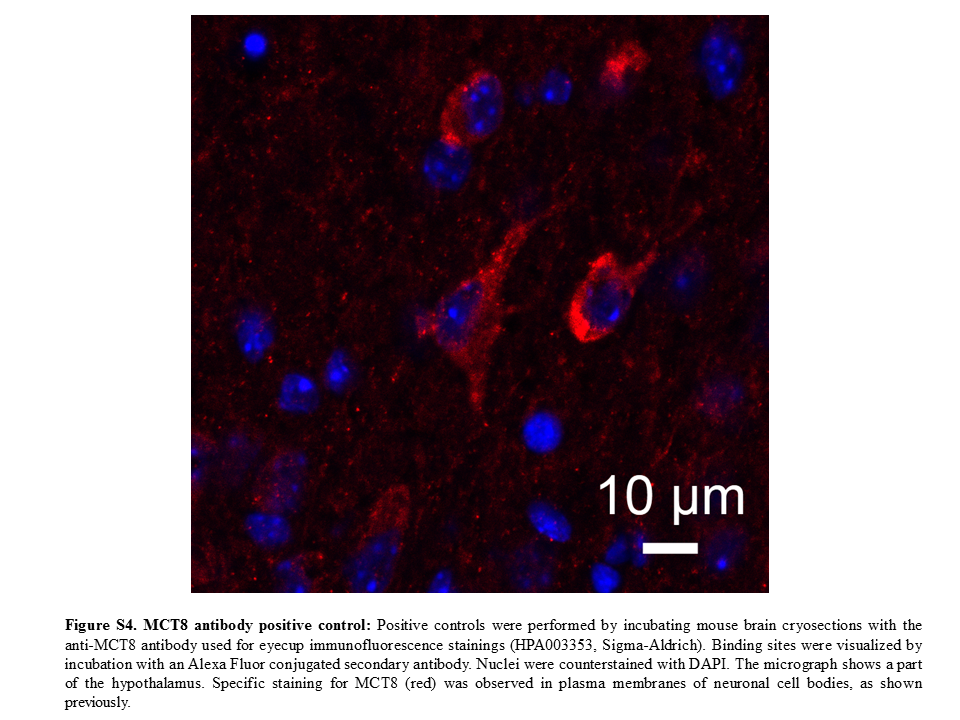

Supplement: Supplementary file 4 [file Image_4.TIF]
